# Secreted Alpha-N-Arabinofuranosidase B Protein Is Required for the Full Virulence of *Magnaporthe oryzae* and Triggers Host Defences

**DOI:** 10.1371/journal.pone.0165149

**Published:** 2016-10-20

**Authors:** Jingni Wu, Yiming Wang, Sook-Young Park, Sang Gon Kim, Ju Soon Yoo, Sangryeol Park, Ravi Gupta, Kyu Young Kang, Sun Tae Kim

**Affiliations:** 1 Division of Applied Life Science (BK21 program), Gyeongsang National University, Jinju, Korea; 2 Plant Molecular Biology and Biotechnology Research Center, Gyeongsang National University, Jinju, Korea; 3 Department of Plant Microbe Interactions, Max Planck Institute for Plant Breeding Research, Cologne, Germany; 4 Korean Lichen Research Institute, Sunchon National University, Suncheon, Korea; 5 Department of Life Science & Environmental Biochemistry, Pusan National University, Miryang, Korea; 6 Molecular Breeding Division, National Institute of Agricultural Sciences, Rural Development Administration, Jeonju, Korea; 7 Department of Plant Bioscience, Life and Industry Convergence Research Institute, Pusan National University, Miryang, Korea; University of Birmingham, UNITED KINGDOM

## Abstract

Rice blast disease caused by *Magnaporthe oryzae* is one of the most devastating fungal diseases of rice and results in a huge loss of rice productivity worldwide. During the infection process, *M*. *oryzae* secretes a large number of glycosyl hydrolase proteins into the host apoplast to digest the cell wall and facilitate fungal ingression into host tissues. In this study, we identified a novel arabinofuranosidase-B (MoAbfB) protein that is secreted by *M*. *oryzae* during fungal infection. Deletion of *MoAbfB* from *M*. *oryzae* resulted in reduced disease severity in rice. Biochemical assays revealed that the MoAbfB protein exhibited arabinofuranosidase activity and caused degradation of rice cell wall components. Interestingly, pre-treatment of rice with the MoAbfB protein inhibited fungal infection by priming defence gene expression. Our findings suggest that MoAbfB secretion affects *M*. *oryzae* pathogenicity by breaking down the host cell wall, releasing oligosaccharides that may be recognized by the host to trigger innate immune responses.

## Introduction

Rice blast disease, caused by the hemibiotrophic fungus *Magnaporthe oryzae*, is one of the most destructive diseases of rice (*Oryza sativa*) [1]. *M*. *oryzae* infection is initiated by conidial attachment to the rice leaf surface, which is followed by the generation of an appressorium structure that applies enormous turgor pressure to penetrate the plant surface and the underlying cells [2]. After penetration, fungal hyphae are surrounded by host cell membranes and grow biotrophically inside host cells, gaining nutrients from the living host cells and entering nearby cells via plasmodesmata [3,4]. Disease symptoms do not appear until the fungus spreads extensively and switches to a necrotrophic growth phase. This hemibiotrophic lifestyle of *M*. *oryzae* suggests that it may evade plant recognition and/or suppress plant immunity to permit its extensive spreading within the rice tissue.

In the early biotrophic infection stage, both the fungus and the host secrete numerous proteins into the apoplastic space, a space outside the plasma membranes of both the fungus and host that allows free diffusion [3, 5–7]. The apoplastic region is the first area of interaction between the host and pathogen, where pathogen-associated molecular patterns (PAMPs) on fungi are recognized by host plasma membrane-localized pattern recognition receptors (PRRs), thereby triggering the host defence response [6,8,9]. Therefore, the characterization of novel elicitor proteins that are secreted from fungi will aid in the understanding of fungal pathogenicity and of the rice–*M*. *oryzae* interaction. To date, only a few secreted protein elicitors from rice blast fungus have been characterized, including PemG1, MoHrip1 and MSP1 [10–12]. PemG1 is a heat-stable protein that was isolated from *M*. *oryzae*. Overexpression of PemG1 in rice confers resistance to blast fungus infection [11]. Pretreatment of rice seedlings with recombinant PemG1 protein triggers systemic acquired resistance in rice through jasmonic acid (JA)-dependent signalling [13]. Another small secreted *M*. *oryzae* protein, MoHrip1, has been characterized as a fungal elicitor. Recombinant MoHrip1 protein can also induce early defence responses, such as hydrogen peroxide production and callose deposition in tobacco, thus conferring enhanced resistance to *M*. *oryzae* [10]. Recently, a novel fungal elicitor, MSP1, was identified and characterized [12]. Upon its secretion by *M*. *oryzae*, MSP1 induces cell death and elicits defence responses in rice [12].

In response to pathogen attack, plants have evolved multiple layers of passive and active defence mechanisms to overcome biotic stresses. Passive defence mechanisms utilize pre-existing structures [14] and preformed anti-pathogen or toxic secondary metabolites [15]. Active defence responses, such as the hypersensitive response (HR) [16], oxidative burst induction [17], and fortification of cell walls [18], are triggered rapidly and directly. The timing and strength of defence response activation determines the extent of host resistance. Such immune responses are triggered by the recognition of PAMPs and damage-associated molecular patterns (DAMPs). Compared to what is known about PAMPs, the DAMP inducers and receptors, which mediate the activation of plant defence signalling, remain poorly characterized. As the main component of plant cell wall structures, oligosaccharides are believed to be the most likely DAMP candidates [19, 20]. During infection, secreted enzymes from pathogens can digest host cell walls to provide carbon and energy sources for pathogen growth [21]. To date, plant wall-associated kinase family proteins (WAKs), which are located in the plant cell membrane, have been proposed as candidate receptors of oligosaccharides in *Arabidopsis* [22,23]. WAK1 is the first well-characterized DAMP receptor in *Arabidopsis* that can recognize degradation products and trigger reactive oxygen species (ROS) production and defence gene expression [22]. In rice, overexpression of OsWAK1 leads to resistance against *M*. *oryzae* infection, and both salicylic acid and JA treatments have been shown to induce OsWAK1 [24].

Our previous *in planta* secretome analysis of rice–*M*. *oryzae* interactions identified over 400 pathogen-secreted proteins, of which glycosyl hydrolase (GH) family proteins were the most conserved, suggesting the possible involvement of these proteins in the rice-*M*. *oryzae* interaction [7]. Here, we identify and characterize an *M*. *oryzae*-secreted protein, MoAbfB, that exhibits arabinofuranosidase and elicitor-like activity. MoAbfB specifically accumulated in invasive fungal structures during rice blast infection, and its secretion was crucial for triggering host immune defences. The arabinofuranosidase activity of MoAbfB induced host cell wall digestion, and the digestion products activated the host defence response. Pre-treatment of rice with the MoAbfB protein induced defence responses that conferred resistance to *M*. *oryzae* infection. Moreover, defence signalling could be activated by released cell wall oligosaccharides, providing new evidence that MoAbfB triggers host defence responses.

## Material and Methods

### Plant materials

Wild-type (*O*. *sativa* L. Jinheung) and *PBZ*1 *promoter*::*GFP* transgenic plants were used in this study [25]. De-hulled rice seeds were imbibed in distilled water at 4°C for 2 days and then planted in field soil in a greenhouse under natural light conditions. Fourth- to fifth-leaf-stage rice plants were used for fungal infection and bombardment assays.

### Fungal stains, transformation, and plant inoculation

*M*. *orzyea* strain KJ201 and KJ301, which were incompatible and compatible to Jinheung rice, respectively, were used in this study. The fungal strain *Δku70*, which causes compatible interaction to Jinheng, were used for generating transgenic fungus. *M*. *oryzae* strains were cultured on rice bran agar medium (25 g/L rice bran, 1 g/L sucrose, and 20 g/L agar) [7] or on complete medium [26] at 25°C under constant light to promote conidial production, as described previously. Conidia were collected from 7-day-old cultures in sterile distilled water and washed twice. Spores were counted using a haemocytometer and resuspended to 5 × 10^5^ conidia/mL in 0.25% gelatin and 0.02% Tween 20. Protoplast polyethylene glycol (PEG) transformation was used for the generation of *M*. *oryzae* mutants, as described previously [27]. Hygromycin- or sulfonylurea-resistant transformants were selected on plates with 50 mg/L of hygromycin B (Sigma—Aldrich, St. Louis, MO, USA) or 100 mg/L of sulfonylurea (Sigma—Aldrich, St. Louis, MO, USA).

Plant incubation, inoculation, and lesion examination were performed as described previously [28]. Briefly, for plant infection assays, fourth- and fifth-leaf-stage rice plants were used for whole plant and leaf sheath inoculations. Disease symptoms were allowed to develop in a humid chamber at 28°C for 3–4 days. Infected leaves were scanned using an HP Precision Scanner at a resolution of 600 dpi (Hewlett Packard, Palo Alto, CA, USA). GFP signals were detected using a LAS-4000 imager (Fujifilm, Tokyo, Japan). Lesion sizes were determined using the APS Assess 2.0 program (APSnet, St. Paul, MN, USA).

### Domain prediction

Amino acid sequence of MoAbfB was used for bioinformatics analysis of protein secretion and domain searching. SignalP 4.1 (http://www.cbs.dtu.dk/services/SignalP/) was used for protein secretion analysis with default setting while NCBI (http://www.ncbi.nlm.nih.gov/) was used for conserved domain prediction.

### Plasmid construction

For protein expression, the MoAbfB (NCBI accession number: MGG_06843) coding sequence was amplified from genomic DNA of wild-type Δku70 strain by PCR without an N-terminal signal peptide but containing *Bam*HI and *Hin*dIII restriction enzyme sites. The amplified fragment was gel-purified (PCR Clean-up System; Promega, Madison, WI, USA) and cloned into the pGEM-T vector (Promega, Madison, WI, USA). The plasmid was digested with *Bam*HI and *Hin*dIII and then subcloned into the pIH1119 expression vector, which encodes a maltose-binding protein (MBP) tag (New England Biolabs, Ipswich, MA, USA).

### Production of recombinant MoAbfB protein and antibody

The recombinant plasmid was transformed into *E*. *coli* (strain BL21 DE3.0), and positive clones were selected on Luria broth (LB) plates containing 100 μg/mL of ampicillin. MBP-tagged MoAbfB protein was purified using an MBP Trap^™^ HP 5-mL column (GE Healthcare, Milwaukee, WI, USA) with a Biologic LP Chromatography System (Bio-Rad, Hercules, CA, USA) according to the manufacturer’s instructions. Recombinant MBP-tagged MoAbfB was collected on the MBP column and cleaved with thrombin (Sigma-Aldrich, St. Louis, MO, USA). Purified recombinant MoAbfB protein showed a molecular weight of 50 kDa. For antibody preparation, purified recombinant MoAbfB protein (200 μg per inoculation) was used to immunize adult female rabbits. The resulting antibodies were purified as previously described [29].

### Leaf treatment with recombinant protein and priming assay

To treat leaves with recombinant MoAbfB protein, 10 μL of a solution containing recombinant protein at the indicated concentration and 0.01% Tween 20 was dropped onto the leaf surface without wounding stress. For the leaf priming test, 0.1 μM MoAbfB in water with 0.01% Tween 20 was sprayed onto rice leaves in a humid chamber 12 hr before fungal infection. Water with 0.01% Tween 20 was used as a control.

### Protein extraction and western blotting analysis

Total and secreted proteins from infected rice leaves were extracted using a PEG fractionation method [30] and a calcium acetate extraction method, as described previously [7,31]. For western blotting analysis, samples containing equal amounts of protein were separated by 12% SDS-PAGE and transferred onto a polyvinylpyrrolidone fluoride (PVDF) membrane using a semidry electrophoretic apparatus (Hoefer, Holliston, MA, USA). The PVDF membrane was blocked for 4 hr at room temperature in 1× TTBS buffer (50 mM Tris-HCl, pH 8.2, 0.1% v/v Tween 20, and 150 mM NaCl) with 7% skim milk and then incubated for 2 hr with primary antibodies against MoAbfB (1:1,000), OsGlu2 (1:5,000), or RuBisCO (1:20,000). The membrane was washed 3 times with 1× TTBS for 15 min. A secondary anti-rabbit IgG antibody conjugated with horseradish peroxidase (diluted 1:10,000 in 1× TTBS) was used for immune detection. The antigen—antibody reaction was incubated for 2 hr, and the resultant reacting proteins were detected by electrochemiluminescence (Perkin Elmer Life Sciences, Boston, MA, USA) using an LAS4000 imaging system (GE Healthcare).

### Arabinofuranosidase activity assay and kinetic analysis

Alpha-l-arabinofuranosidase (AbfB) enzymatic activity was measured using *para*-nitrophenyl-alpha-l-arabinofuranoside (PNP-A; Sigma-Aldrich) as a substrate. Reaction mixtures of 500 μL contained 1 mM PNP-A in 50 mM sodium phosphate buffer (pH 7.0) and 0.4 mg/mL of purified MoAbfB protein. The reaction was performed at 45°C for 30 min and was stopped by adding 1 mL of sodium carbonate. The absorbance was measured at 415 nm with a DU 800 Spectrophotometer (Bio-Rad). AbfB activity was determined using a standard curve generated with a pNP standard, which was assayed under similar conditions. The K_m_ and V_max_ values were calculated from the initial rate of pNP liberation using Prism 5.0 software (GraphPad, San Diego, CA, USA). Protein concentrations were determined by the Bradford method.

### Transient gene expression by particle bombardment

Transgenic rice harbouring the *PBZ*1 *promoter*:*GFP* reporter was chosen for bombardment assays. *PBZ*1 *promoter*:*GFP* was used as a defence marker in this study, which showed successful activation upon pathogen infection and programmed cell death [25]. The leaf sheath tissue of PBZ1 *promoter*::*GFP* transgenic rice plants at the fourth- to fifth-leaf stage was sliced with a razor blade and laid onto half-strength Murashige and Skoog (MS) medium in darkness for 1 hr at 28°C [8]. A constructed expression vector (2 μg) was attached along with 20 μL of 0.1 M spermidine and 40 μL of 2.5 M calcium chloride to the tungsten M-17 microcarrier. All bombardments were conducted using a Biolistic PDC-1000/He system (Bio-Rad). Bombarded rice leaf sheaths were incubated on half-strength MS medium for another 48 hr. The fluorescent signal was then detected using a FLUOVIEW FV1000 confocal laser scanning microscope (Olympus, Tokyo, Japan). The GFP and mCherry fluorophores were excited at wavelengths of 395 nm and 543 nm, respectively, and fluorescence signals were observed at 450–490 nm and 587–625 nm, respectively.

### Targeted deletion of the *MoAbfB* gene and Δ*MoAbfB* complementation in *M*. *oryzae*

To generate *MoAbfB* gene deletion mutants, a knock-out vector was constructed. Based on the *MoAbfB* gene sequence in the *M*. *oryzae* genome, 1.3 kb of the 5’ flanking sequence and 1.3 kb of the 3’ flanking sequence were amplified using the primer pairs MoAbfB _5F_*Kpn*I and MoAbfB _5R_*Xho*I for the 5’ flanking region and MoAbfB _3F_*Hin*dIII and MoAbfB _3R_*Spe*I for the 3’ flanking region (S1 Table) from wild-type *Δku70* genomic DNA. The 1.4-kb hygromycin (HYG) marker cassette was amplified from pBCATPH [32] using the primers HygB_F_*Xho*I and HygB_R_*Hin*dIII. These 3 amplicons were cloned into the pGEMT-Easy vector (Promega, WI, USA). All clones were verified by sequencing. A knock-out construct was generated by ligating the 5’ flanking region (*Kpn*I-*Xho*I fragment), HYG cassette (*Xho*I-*Hin*dIII) and 3’ flanking region (*Hin*dIII-*Spe*I) between the *Kpn*I and *Spe*I sites of pBC SK+ (Stratagene, La Jolla, CA, USA). Wild-type fungal protoplasts were directly transformed with the clones, as previously described [33]. Putative gene deletion mutants were screened, and the candidate mutants were subsequently purified by isolating single conidia. Southern blotting analysis was performed to confirm the deletion mutants, as previously described [34].

Complementation of the Δ*MoAbfB* deletion was achieved by amplifying a 4.3-kb fragment, containing the *MoAbfB* open reading frame (ORF) along with 1.5 kb of the 5’flanking region and 1.3 kb of the 3’ flanking region, from wild-type genomic DNA using the MoAbfB _5F_*Kpn*I and MoAbfB _3R_*Spe*I primers. The purified 4.3-kb complementation construct was co-transformed with the PII99 plasmid [35] carrying a gene conferring resistance to geneticin into Δ*MoAbfB* protoplasts. Putative complemented transformants were selected on TB3 plates supplemented with 800 ppm geneticin. After genetic purification by single conidium isolation, complemented clones were confirmed by the detection of *MGG_06843T0* gene expression by RT-PCR.

### Phenotypic assays of the Δ*MoAbfB* mutant and complemented strain

Vegetative growth, conidiation, conidial germination, appressorium formation, and infection assays with rice seedlings were conducted as previously described [33].

### Semiquantitative RT-PCR

Total RNA was isolated using a Plant Mini RNA Kit (Qiagen, Valencia, CA, USA), and cDNA was synthesized using the SuperScript III First-Strand Synthesis System (Invitrogen, Carlsband, CA, USA). The PCR products were amplified using specific primer sets (S1 Table). The PCR programme began with a 95°C incubation for 2 min that was followed by 30 cycles of 95°C for 30 s, 55°C for 30 s, and 72°C for 40 s. The PCR products were separated on a 1% agarose gel containing ethidium bromide. The transcription abundances of each of the 3 replicates were imaged using a LAS-4000 (Fujifilm, Tokyo, Japan).

### Scanning electron microscopy

The MoAbfB protein-treated rice leaves were fixed in a fixation solution (2.5% glutaraldehyde and 0.1 M sodium cacodylate, pH 7.4) for 12 hr on ice and were then dehydrated in a graded ethanol series (25%, 50%, 75% 95%, and 100% ethanol, 30 min in each step). The dehydrated materials were critical-point dried using liquid CO_2_ and then mounted onto metal stubs (Semidri-795; Tousimis, Rockville, MD, USA). Mounted samples were shadowed with gold before observation by scanning electron microscopy (XL30 S FEG; Philips, Eindhoven, The Netherlands).

### Glycosyl composition analysis

To analyse the sugar composition of recombinant MoAbfB-digested cell wall extracts, samples were freeze-dried and resuspended in water before being passed through a 5-mL column packed with mixed-bed exchange resin. The flow-through eluate was freeze-dried and then passed through a C18 Sep-Pak (Waters, Maidstone, Kent, UK). Both the water fraction and the methanol fraction were collected and analysed. The water fraction was lyophilized and prepared for compositional analysis and matrix-assisted laser desorption ionization-time of flight mass spectrometry (MALDI/TOF-MS) analysis. MALDI/TOF-MS was performed in both reflector-positive and -negative ion modes. All spectra were obtained using an AB SCIEX TOF/TOF^™^ 5800 (Applied Biosystems, Foster City, CA, USA). The methanol fraction was used for glycosyl composition analysis based on combined gas chromatography/MS (GC/MS) analysis of the per-O-trimethylsilyl (TMS) derivatives of monosaccharide methyl glycosides produced from the sample by acidic methanolysis. This analysis was conducted by the Complex Carbohydrate Research Center (University of Georgia, Athens, GA, USA).

## Results

### *M*. *oryzae* secretes MoAbfB during infection

**Fig 1:**
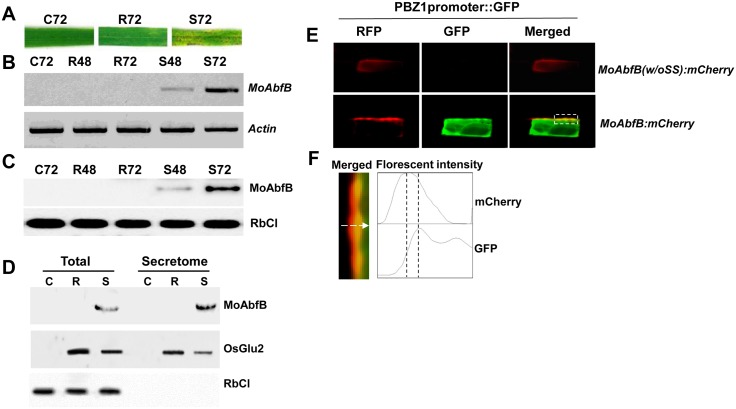
Expression and secretion of MoAbfB during host infection by *M*. *oryzae*. (A) Typical infection phenotype of control (C), incompatible (R), and compatible (S) interactions at 72 hr post *M*. *oryzae* infection. (B) Transcriptional and (C) translational expression of MoAbfB in rice leaves, as confirmed by semi-quantitative RT-PCR and western blotting analysis, respectively, in R and S combinations after 48 and/or 72 hr. Rubisco large subunit (RbCl) was set as loading control (D) Western blotting analysis of MoAbfB, OsGlu2, and Rubisco expression in total protein extracts (total) and secreted protein fractions (secretome) extracted from *M*. *oryzae*-infected rice leaves. Samples were collected at 72 hr. (E) Transient expression of MoAbfB-mCherry and MoAbrB(w/oSS)-mCherry constructs in sheath cells of transgenic rice harbouring a *PBZ*1 *promoter*::*GFP* reporter. Florescent signals were detected at 48 hr post-particle bombardment. (F) Close-up view and quantification of the mCherry and GFP fluorescent signals in the direction indicated by the arrow.

Previous studies of the *in planta* secretome during rice–*M*. *oryzae* interaction identified a number of GHs that were expressed during the fungal infection process, including an *M*. *oryzae* arabinofuranosidase B protein (MoAbfB) [7]. To investigate the involvement of MoAbfB in fungal pathogenicity, we first measured the expression of *MoAbfB* in rice leaves using RT-PCR and immunoblotting analysis under typical control, incompatible and compatible interactions between rice-*M*. *oryzae* (Fig 1A). MoAbfB accumulated in rice leaves at both the transcriptional and translational levels only during compatible fungal infection, as no accumulation was observed during incompatible infection (Fig 1B and 1C). Bioinformatic analysis revealed that MoAbfB is a predicted secretory protein containing an N-terminal 25-amino-acid signal peptide (S1 Fig). To confirm the apoplastic localization of MoAbfB, immunoblotting analysis was performed using total and secreted protein fractions (Fig 1D). Secreted proteins were extracted from mock-infected and fungus-infected rice leaves as previously described [7,29]. OsGlu2 is a defence marker that is induced by *M*. *oryzae* infection and acts as a secreted marker protein in the apoplastic region [7]. OsGlu2 was detected in both the total and secreted protein fractions during both incompatible and compatible interactions. Furthermore, the intracellular protein RuBisCO (ribulose-1,5-bisphosphate carboxylase/oxygenase) was detected only in total fractions and not in secreted protein fractions, indicating the purity of the isolated apoplastic proteins from *M*. *oryzae*-infected rice leaves. These results indicate that MoAbfB is secreted from *M*. *oryzae* into the apoplastic space during *in planta* infection.

### Secretion of MoAbfB triggers host defence

Elicitor proteins are well-known to induce defence responses in host cells. To test whether MoAbfB exhibits elicitor activity, promoter induction analyses were performed by transient expression of MoAbfB in transgenic rice harbouring the *PBZ*1 *promoter*::*GFP* reporter, which is commonly used as a defence marker in rice [25]. The MoAbfB sequence with and without a signal sequence (w/o SS) was fused to an mCherry reporter and placed under the maize ubiquitin (*ZmUbi*) promoter. The mCherry signal was detected 48 hr after the transient expression of both constructs. However, the green fluorescent protein (GFP) signal, indicating the induction of the *PBZ*1 promoter, was detected only in response to the expression of MoAbfB harbouring the signal peptide (Fig 1E). A close-up view of the merged signals demonstrates that the fluorescence signal from mCherry partially overlapped but extended beyond the cytoplasmic GFP signal (Fig 1F). This result indicates that the MoAbfB-mCherry fusion protein accumulated outside of the rice plasma membrane (Fig 1F).

### *MoAbfB* is required for the virulence of *M*. *oryzae*

**Fig 2:**
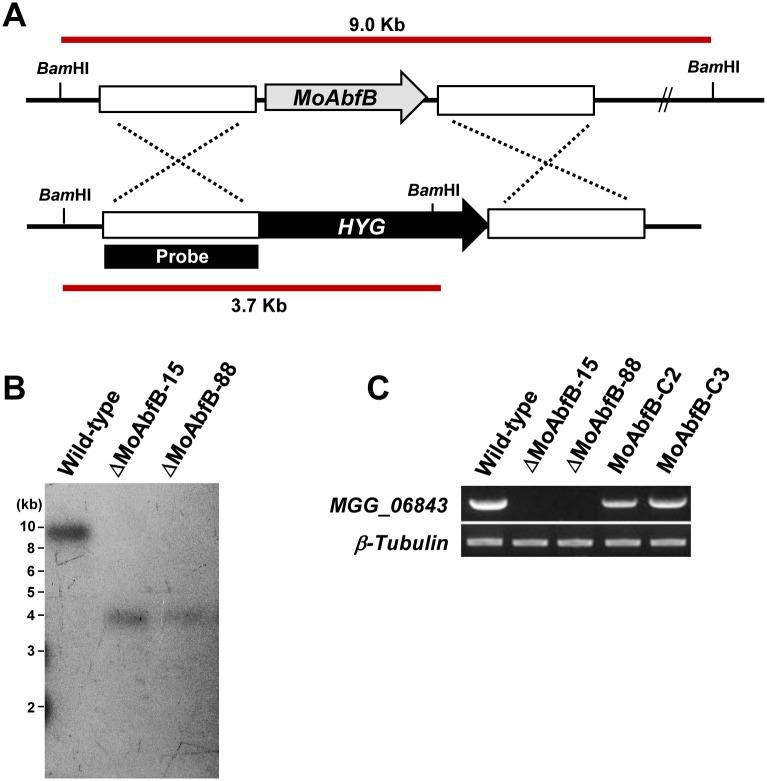
Generation of the Δ*MoAbfB* mutant and complemented strain. (A) Gene disruption strategy for MoAbfB (MGG_06843T0). HYG, hygromycin B phosphotransferase marker gene cassette. (B) Genomic DNA samples from a wild-type strain and 3 Δ*MoAbfB* mutants were digested with *Bam*HI and probed with the 5’-flanking 1,284 bp of the MoAbfB gene. (C) RT-PCR to confirm the loss and recovery of the MoAbfB transcript.

To determine the role of *MoAbfB* in *M*. *oryzae* pathogenicity, a gene deletion mutant (*ΔMoAbfB)* was generated along with its complement, MoAbfBc (Fig 2 and Table 1). Deletion mutants lacking the *MoAbfB* gene exhibited significant reductions of vegetative growth and conidiation compared with the wild-type. However, we observed no significant difference in germination and appressorium formation between the mutants and the wild-type (Table 2). The defective phenotypes associated with deletion of the *MoAbfB* gene were also rescued by complementation (Table 2). These results suggest that *MoAbfB* is related to fungal growth and conidiogenesis.

**Table 1:** Strains used in this study.

Number	Strain Name	Strain type	Usage
1	KJ201	incompatible	Induction of incompatible interactions
2	KJ301	compatible	Induction of compatible interactions
3	*ΔKu70*	compatible	Generating fungal mutant
4	*Δ06843–15*	compatible	*MoAbfB* knockout strain
5	*Δ06843–88*	compatible	*MoAbfB* knockout strain
6	Δ06843-15-C2	compatible	*MoAbfB* complementation strain
7	Δ06843-15-C3	compatible	*MoAbfB* complementation strain

**Table 2:** Characterization of Δ*MoAbfB* mutants and complemented strains.

Strain	Growth (mm) ^a^	Conidiation (10^4^/mL) ^b^	Germination (%) ^c^	App. Formation (%) ^d^
Wild-type	78.0 ± 1.0^A^ ^e^	53.5 ± 9.6^A^	95.3 ± 0.6^A^	98.0 ± 1.0^A^
*ΔMoAbfB-1*	72.7 ± 2.5^B^	38.4 ± 6.8^B^	95.3 ± 0.6^A^	97.3 ± 1.2^A^
*ΔMoAbfB-2*	72.2 ± 2.5^B^	41.3 ± 8.3^B^	94.7 ± 0.6^A^	98.7 ± 0.6^A^
MoAbfBc-1	74.0 ± 1.7^AB^	47.0 ± 3.1^AB^	95.3 ± 0.6^A^	97.7 ± 1.2^A^
MoAbfBc-2	77.0 ± 1.7^AB^	44.1 ± 9.5^AB^	95.0 ± 1.0^A^	98.7 ± 0.6^A^

Data are presented as the means ± SD from 3 independent experiments. The same letters in a column indicate the absence of a significant difference.

^a^ Vegetative growth was measured at 7 days post-inoculation on complete agar medium.

^b^ Conidiation was measured by counting the number of conidia collected with 5 mL of sterilized distilled water from 7-day-old V8 Juice agar plates. Data are presented as the means ± SD from 3 independent experiments.

^c^ Percentage of conidial germination on hydrophobic surfaces was measured under a light microscope using conidia harvested from 7-day-old V8 juice agar plates.

^d^ Percentage of appressorium formation on hydrophobic surfaces was measured using conidia harvested from 7-day-old V8 juice agar plates.

^e^ Tukey’s test was used to determine significance at the 95% probability level. The same letters in a column indicate the absence of a significant difference.

**Fig 3:**
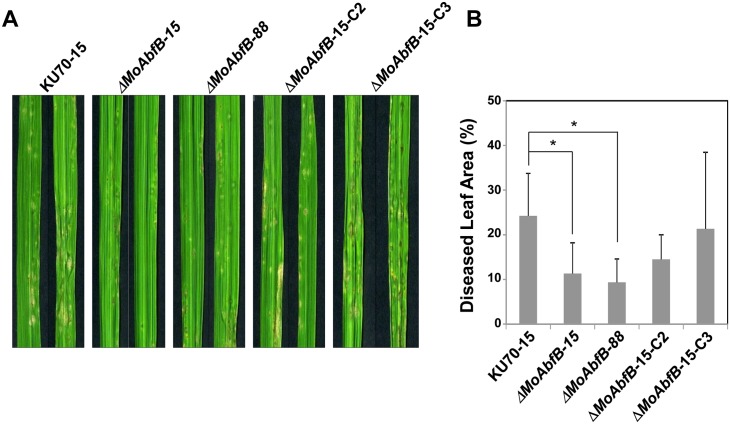
Assay for pathogenicity. (A) Conidial suspensions (5 × 10^5^ conidia/mL) of the indicated isolates were sprayed on 3–4 leaf-stage rice leaves. Photographs were taken 7 days after inoculation. (B) Percentage of infected areas of seedlings infected with wild-type fungi, Δ*MoAbfB* mutants, or MoAbfBc complemented strains. Error bars indicate standard deviation from the mean of 3 independent experiments (*, *P* < 0.05; Tukey’s test).

In spray-inoculation tests, the *ΔMoAbfB* mutant exhibited significant reduction of virulence, whereas the wild-type and complemented transformants caused typical susceptible-type spreading lesions (Fig 3A and 3B). This result suggests that the *MoAbfB* gene is involved in pathogenesis. The defective phenotypes resulting from deletion of the *MoAbfB* gene were also rescued by complementation.

### MoAbfB exhibits alpha-l-arabinofuranosidase activity

**Fig 4:**
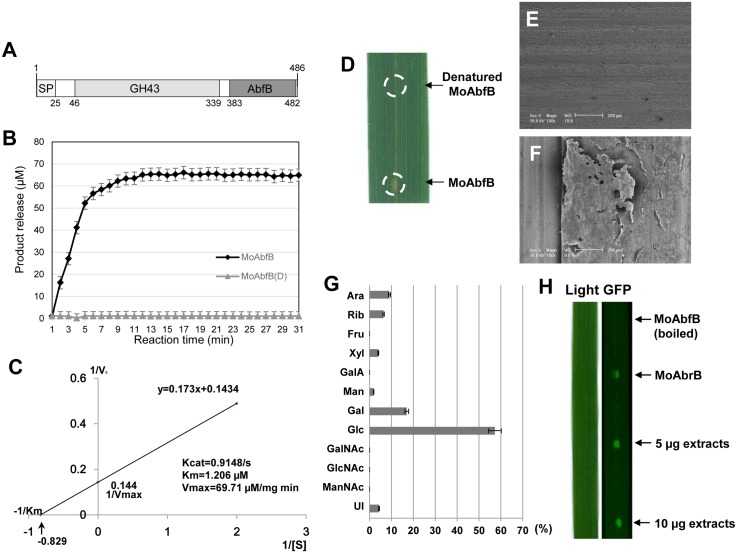
Recombinant MoAbfB protein degrades cell wall structures. **(A)** Schematic diagram of signal peptide and conserved domain in MoAbfB. SP, signal peptide; GH43, glycosyl hydrolase family 43 domain; AbfB, Alpha-L-arabinofuranosidase B domain. (B) Nonlinear regression plot of time-dependent MoAbfB and denatured MoAbfB (MoAbfB(D)) release of ρNPA (pH 7.0). (C) Enzyme kinetics assay of recombinant MoAbfB protein. A linear regression plot constructed using GraphPad Prism 5.04 (GraphPad Software Inc., 1992–2010) was created using the Michaelis—Menten saturation kinetic constants for the ability of recombinant MoAbfB to produce ρNPA. (D) Morphology of MoAbfB-digested rice leaf surface (with 5 μg of recombinant MoAbfB protein) after 72 hr of treatment. (E–F) SEM images of MoAbfB protein-digested host cell walls. (E) Boiled enzyme-treated rice leaves exhibited a clear and complete surface structure. (F) Damaged cell surfaces and degraded cell wall matrices caused by MoAbfB treatment. (G) Sugar composition of MoAbfB-digested cell wall extracts. ‘Ara’ = arabinose; ‘Rib’ = ribose; ‘Fuc’ = fucose; ‘Xyl’ = xylose; ‘UI’ = unidentified sugar; ‘GalA’ = galacturonic acid; ‘Man’ = mannose; ‘Gal’ = galactose; ‘Glc’ = glucose; ‘GalNAc’ = N-acetylgalactosamine; ‘GlcNAc’ = N-acetylglucosamine; ‘ManNAc’ = N-acetylmannosamine. (H) Activation of cell death by MoAbfB. Boiled MoAbfB protein (5 μg) (1) and native MoAbfB protein (5 μg) (2) were used as negative and positive controls, respectively; 5 μg (3) and 10 μg (4) of oligosaccharides (glucose equivalent) were dropped onto *PBZ*1 *promoter*::*GFP* rice leaves. GFP signals were detected after 72 hr.

Based on domain prediction, MoAbfB was found to contain a GH43 subfamily domain (amino acids 46–339) and an alpha-l-arabinofuranosidase B (AbfB) domain (amino acids 383–482) (Fig 4A), suggesting that MoAbfB might be a secreted protein exhibit arabinofuranosidase enzyme activity. Therefore, we overexpressed and purified recombinant MoAbfB protein. In a biochemical activity assay using purified recombinant MoAbfB protein, a 95% confidence level for the reaction was reached at 10 min (Fig 4B), with a maximum enzyme substrate specific activity (V_max_) of 69.71 μM/mg min^-1^ and a K_m_ value of 1.206 μM (Fig 4C). Moreover, the denatured MoAbfB protein presents no enzyme activity, which set as control (Fig 4B). These results indicate that MoAbfB exhibits specific arabinofuranosidase activity.

### Cell wall digestion by MoAbfB triggers host immune responses

As a pathogen-secreted cell wall-degrading enzyme, secreted MoAbfB may be utilized by *M*. *oryzae* to increase its invasion efficacy. We tested this hypothesis by exogenously treating rice leaves with recombinant MoAbfB to measure its cell wall degradation ability. A clear and complete surface structure was observed for the buffer-only and boiled-MoAbfB controls (Fig 4D and 4E). In contrast, MoAbfB treatment resulted in a brown colour and a damaged cell surface (Fig 4D and 4F). A close-up view of the damaged structure revealed that the MoAbfB protein had degraded the rice cell wall matrix (Fig 4F). The oligosaccharides that were released from the leaves by MoAbfB digestion were later extracted and subjected to composition analysis, which revealed them to be 9.1% arabinose, 6.4% ribose, 4% xylose, 1.9% mannose, 17% galactose, and 57.3% glucose (Fig 4G).

It has previously been reported that oligosaccharides released from the *Arabidopsis* cell wall function as DAMP signals during host-microbe interactions [22,24]. To test whether the MoAbfB-released oligosaccharides work as DAMP signals in rice, cell wall digests were extracted and dropped onto leaf surfaces of the *PBZ*1 *promoter*::*GFP* plant. Importantly, the *PBZ*1 promoter-controlled GFP signal was activated after dropping recombinant but not denatured MoAbfB protein onto leaf surfaces (Fig 4H). Similarly, GFP signals were detected in leaves following treatment with 5 μg or 10 μg (glucose equivalent) of oligosaccharide extracts. Based on this result, we hypothesize that the released oligosaccharides (Fig 4D) may act as elicitors to trigger immunity in rice.

### Priming with MoAbfB suppresses fungal infection

**Fig 5:**
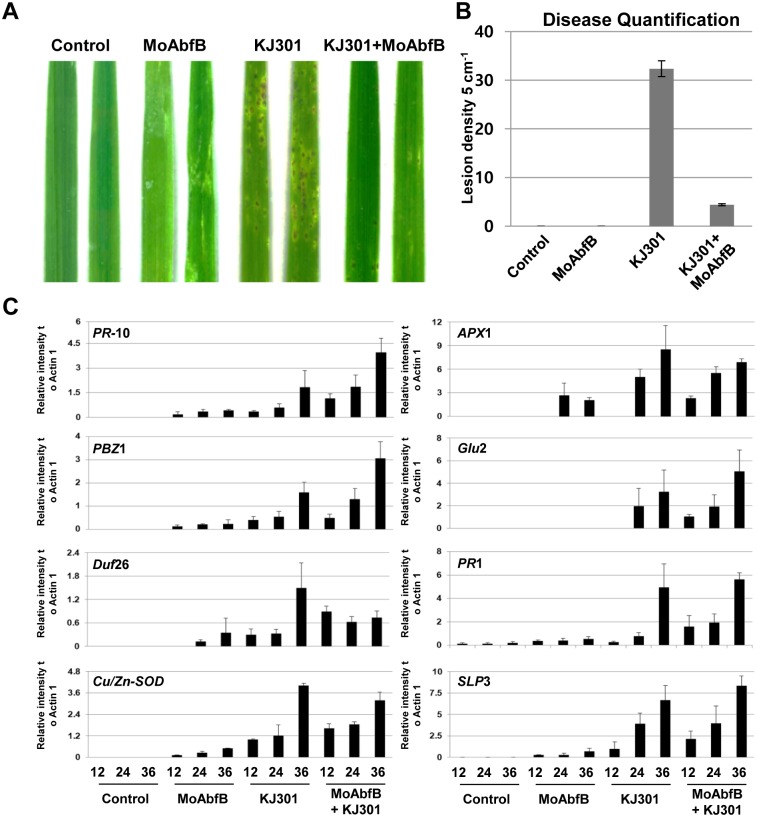
Pre-treatment with MoAbfB suppresses the virulence of *M*. *oryzae* and triggers a host immune response. (A) Fungus-infected lesions on rice leaves pre-treated with 0.1 μM MoAbfB protein 12 hr prior to inoculation and measured at 72 hpi. (B) Lesion areas were quantified using the APS Assess 2.0 program. Error bars indicate the standard deviation from the mean of 3 independent experiments. (C) Expression of ROS and PR genes detected by RT-PCR.

The expression of defence-related genes leads to the suppression of pathogen infection. To investigate whether MoAbfB-triggered host immune responses lead to the suppression of *M*. *oryzae* infection, we used a virulent strain of *M*. *oryzae* (KJ301) to infect MoAbfB-primed rice leaves. No visible cell death occurred in leaves pre-sprayed with 0.1 μM MoAbfB alone (Fig 5A). However, significantly increased resistance to the virulent *M*. *oryzae* strain was observed in MoAbfB-primed leaves (KJ301+MoAbfB in Fig 5A), and the percentage of diseased leaf area was dramatically reduced (Fig 5B). To confirm that the suppression of fungal infection was caused by the MoAbfB-triggered immune response, the transcriptional expression levels of rice defence-related genes and ROS-related genes were measured. As shown in Fig 5C, MoAbfB protein alone was able to trigger the activation of most of the tested defence genes at 12 or 24 hpi. ROS-related genes, such as *Cu/Zu-SOD* and *Apx1*, and pathogenesis related (PR) genes, such as *PR-10*, *PR-1*, and *PBZ1*, were expressed earlier and more strongly in MoAbfB-primed leaves than in KJ301-inoculated leaves (Fig 5C), indicating that early activation of PR genes by MoAbfB can suppress pathogen growth.

## Discussion

The plant cell wall, which is composed of polysaccharide networks, functions as the first line of defence against pathogen infection [36]. Therefore, perforation of the cell wall is a primary task of a filamentous fungus for the successful invasion of host cells. To achieve perforation, cell wall-degrading enzymes (CWDEs) such as endo-β-1,4-d-xylanase are secreted by the fungus into the apoplastic region during the early infection process [37]. The α-L-Arabinofuranosidase family protein is conserved between different fungal species, which exhibits in vitro Arabinofuranosidase acvity [38–41]. Those fungal proteins could classified into two subgroups (S2A Fig), and the protein sequence are more conserved in the GH43 domain among the subgroup I Arabinofuranosidases, which contains MoAbfB (S2B Fig). Previously, knockdown of secreted xylanases in *M*. *oryzae* was shown to reduce its pathogenicity in rice [42]. MoAbfB was identified in a previous *in planta* secretome analysis, which revealed that MoAbfB is secreted during the fungal infection process [7]. It was also shown that the expression level of arabinofuranosidase affects the cell wall structure of *Arabidopsis* [43] and rice [44] through modification and breakdown of host cell wall components. This finding is corroborated by biochemical results for arabinofuranosidase enzyme activity and cell wall degradation in our study (Fig 4).

Our results have shown that absence of *MoAbfB* in *M*. *oryzae* also affects fungal growth and conidiation (Table 2), suggesting *MoAbfB* plays a role in fungal development. It was previously reported that protein secretion of *M*. *oryzae* is essential for mycelial growth, conidial production and host infection [45]. The secreted effectors, such as MC69, not only related with fungal pathogenesis, but also conidiation [46]. Interestingly, supply of various carbon sources in growth medium affects the morphology of *M*. *oryzae* growth [47]. Therefore, it may be possible that secretion of CWDEs affect fungal growth and/or pathogenicity via regulating environment carbon sources. However, the growth morphology and pathogenicity may not be correlated. One interesting result of the present study is that pretreatment with recombinant MoAbfB at a low dosage had a priming effect on the rice immune defence. This priming effect included activation of defence-related proteins and the suppression of pathogen infection. By definition, priming in plants is a process in which a faster and/or stronger defence response is triggered without actually beginning the defence responses prior to the stress condition [48]. Priming is important in plants, as the actual defence responses can be activated without suffering reduced fitness or yield [49]. This priming effect has previously been observed for pre-treatment of rice leaves with the *M*. *oryzae* elicitors PemG1, MoHirp1 and MSP1 [10–12]. In this study, MoAbfB primed immune responses by the activation of ROS and rice PR genes (Fig 5), which is consistent with previous results showing that rice triggers a priming effect to protect itself from pathogen infection through early recognition of and defence activation by fungus-secreted elicitor proteins [10,12,13].

Previously, an endocellulase from *Rhizoctinia solani*, EG1, was demonstrated to inducing immune responses in maize due to its protein structure, without exhibiting any enzymatic activity [50]. However, in this study, the MoAbfB protein was observed to function differently. Specifically, denatured MoAbfB protein exhibited no activation ability, whereas active MoAbfB that was able to digest the cell wall into oligosaccharide fragments directly triggered the host defence response (Fig 4G). This result indicates that MoAbfB enzymatic activity is required for its elicitor activity. Moreover, oligosaccharides that are released from arabinofuranosidase degraded cell walls are involved in host defence elicitation (Fig 4H). Similarly, oligosaccharides act as endogenous elicitors and induce host innate immunity via recognition by membrane receptors [19]. Experimental evidences have suggested that different plant hosts possess similar systems that are able to recognize host-derived oligosaccharides to trigger immune responses [17,51]. The oligosaccharides released from MoAbfB-digested leaves contained sugars such as glucose, galactose, arabinose, and ribose (Fig 4H). There are two possible explanations for how MoAbfB triggers immune responses in hosts. First, the release of particular oligosaccharides by pathogen-derived hydrolases may be recognized by plant membrane-localized receptors, possibly WAK family receptors, to prime DAMP-triggered immunity. Previously, it was shown that oligosaccharides have the ability to induce host defence responses [52]. The second possibility is that the total sugar content or ratio among particular oligosaccharides in the apoplastic region is shifted, and it is the shift that is sensed to trigger an immune response. In *Arabidopsis*, a high ratio of sucrose to hexose triggers the accumulation of anthocyanin, a protective agent [53]. Therefore, investigation of the mechanisms behind the oligosaccharide-triggered immunity observed in the present study represents an interesting subject for future studies. Moreover, the MoAbfB showed strong enzyme activity even at high temperature (45°C), and with a minimum amount of 1 μg showed successful activation of host defense (data not shown). The recombinant MoAbfB protein as well as the cell wall debrides may be used for potential antifungal drug designing in future.

To induce host defence responses against attacking pathogens, a series of signal transduction pathways are activated. Phytohormones such as jasmonic acid (JA) and salicylic acid (SA) are important for defence signal regulation. In rice, a positive correlation between JA and SA and the induction of immune responses has been observed [54]. However, as a monocotyledonous plant, rice contains a high level of endogenous SA, although JA may play a more important role in antifungal defences [55]. In this study, both JA-dependent defence genes (*PBZ*1 and *PR*-10) and SA-dependent defence genes (*PR*1) were upregulated in MoAbfB-primed rice leaves (Fig 5C), suggesting that both JA- and SA-dependent signalling pathways are associated with MoAbfB-induced defence signalling in rice. Similar to SA and JA signalling, ROS signalling is also known to be involved in plant defence mechanisms against bacterial and fungal pathogens [17]. *Cu/Zn-SOD*, *APX*1 and *APX*2 reportedly play roles in the detoxification of ROS and in defence responses. High expression levels of these ROS-related genes in MoAbfB pre-primed leaves indicated that ROS production was induced by MoAbfB treatment. Collectively, our functional analysis of *M*. *oryzae*-secreted proteins provides new clues about the apoplastic interactions of rice and *M*. *oryzae* and supports a potential role for oligosaccharides as DAMP signals that trigger the host immune response.

## Supporting Information

S1 Fig (A) Prediction of signal peptide in MoAbfB by SignalP 4.1. (B) Prediction of conserved domain in MoAbfB sequence by NCBI.
(TIF)

S2 Fig (A) Phylogenetic tree of Arabinofuranosidases in different fungal species. Number indicates the protein identity to MoAbfB. (B) Alignment of protein sequences of SubgroupI Arabinofuranosidases. *S*. *charteusis*, (*Streptomyces chartreusis*, NCBI BAA90772); *S*. *avermitlis*, (PDB 3AKF_A); *H*. *Insolens*, (*Humicola insolens*, NCBI AIM56896.1); *Chaetomium* (*Chaetomium* sp. CQ31, NCBI AFU88757); *A*. *nidulans* (*Aspergillus nidulans*, NCBI XP659175.1); *T*. *reesei*, *(Trichoderma reesei*, NCBI XP_006967945.1); *T*. *konigii*, (NCBI AAA81024.1) *A*.*niger*. (*Aspergillus nidulans*, NCBI XP_001396769.1)
(TIF)

S1 Table Primers used in this study. (DOCX)
