# Supplementary figures and images for: Secreted Alpha-N-Arabinofuranosidase B Protein Is Required for the Full Virulence of Magnaporthe oryzae and Triggers Host Defences
Source: PLoS One. 2016 Oct 20;11(10):e0165149. doi: 10.1371/journal.pone.0165149 (PMC5072668; doi:10.1371/journal.pone.0165149)

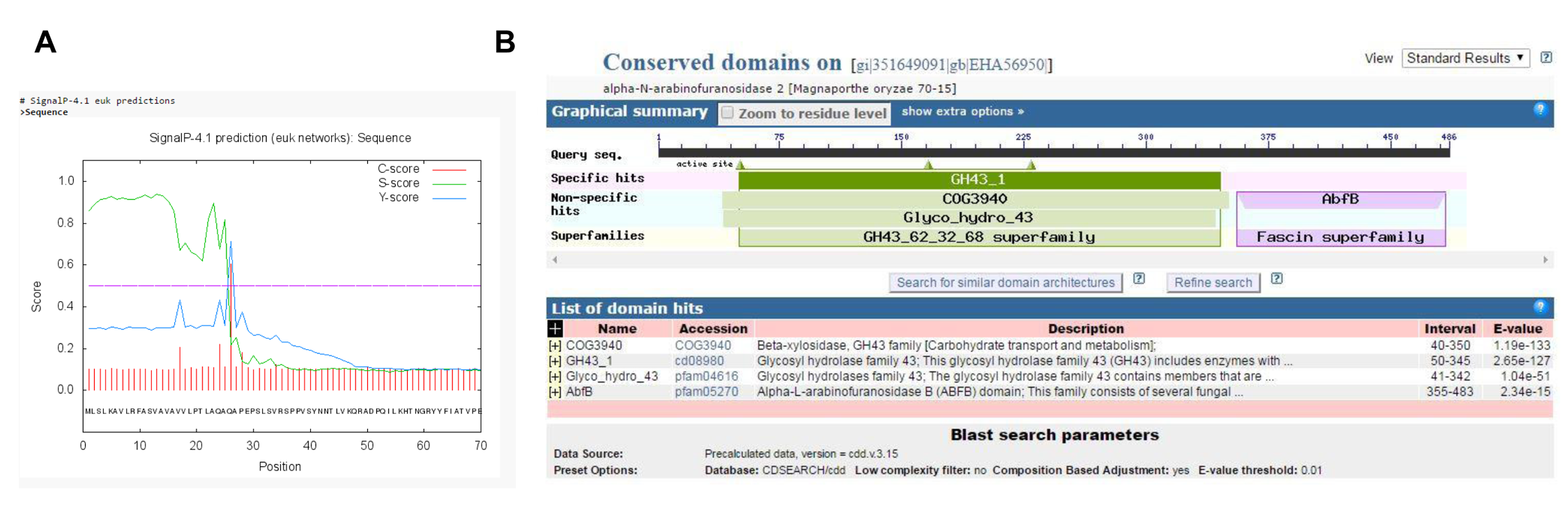

Supplement: S1 Fig — (A) Prediction of signal peptide in MoAbfB by SignalP 4.1. (B) Prediction of conserved domain in MoAbfB sequence by NCBI. (TIF) [file pone.0165149.s001.tif]

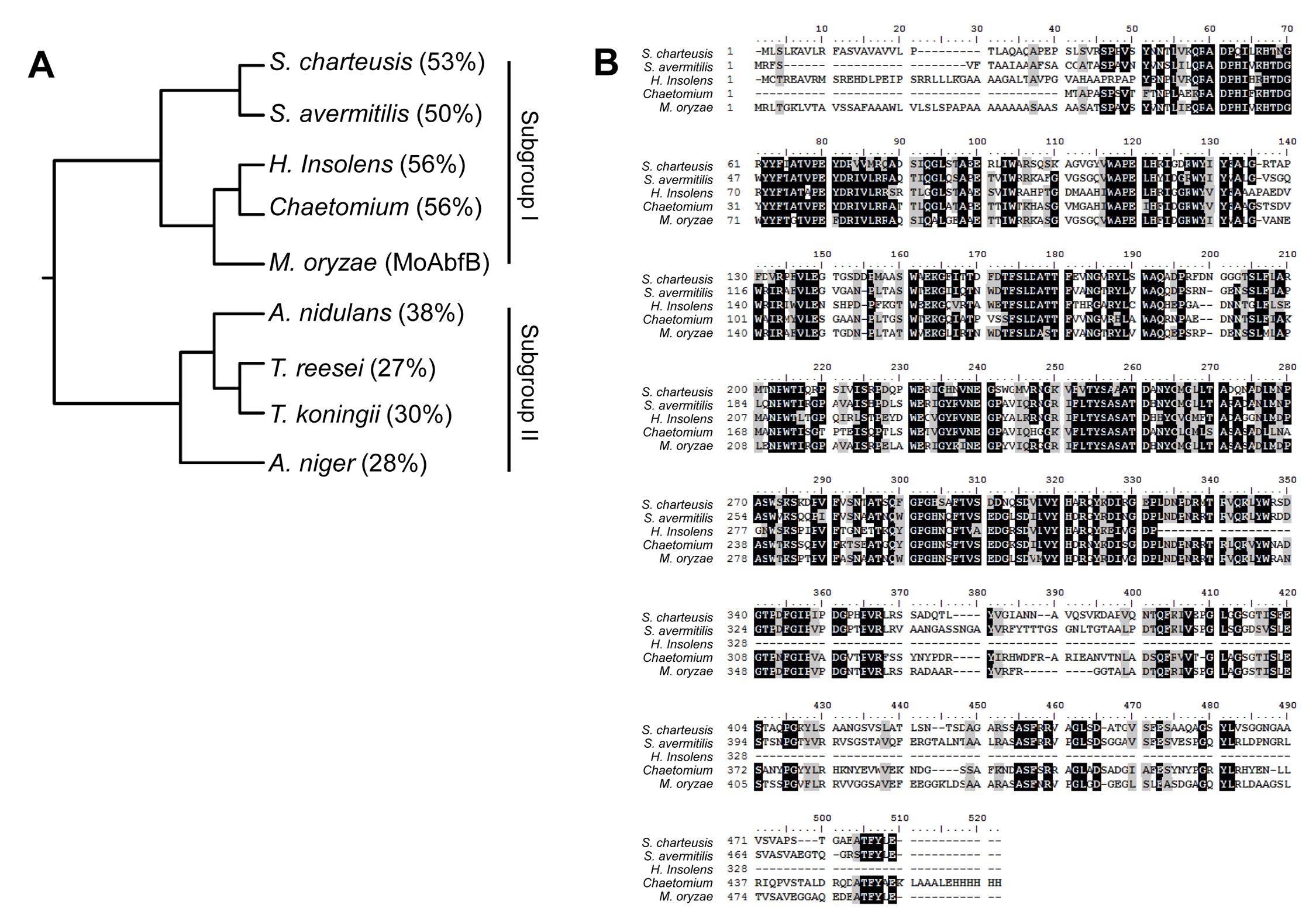

Supplement: S2 Fig — (A) Phylogenetic tree of Arabinofuranosidases in different fungal species. Number indicates the protein identity to MoAbfB. (B) Alignment of protein sequences of SubgroupI Arabinofuranosidases. S. charteusis, (Streptomyces chartreusis, NCBI BAA90772); S. avermitlis, (PDB 3AKF_A); H. Insolens, (Humicola insolens, NCBI AIM56896.1); Chaetomium (Chaetomium sp. CQ31, NCBI AFU88757); A. nidulans (Aspergillus nidulans, NCBI XP659175.1); T. reesei, (Trichoderma reesei, NCBI XP_006967945.1); T. konigii, (NCBI AAA81024.1) A.niger. (Aspergillus nidulans, NCBI XP_001396769.1) (TIF) [file pone.0165149.s002.tif]
