# Supplementary material for: Secreted Alpha-N-Arabinofuranosidase B Protein Is Required for the Full Virulence of Magnaporthe oryzae and Triggers Host Defences
Source: PLoS One. 2016 Oct 20;11(10):e0165149. doi: 10.1371/journal.pone.0165149 (PMC5072668; doi:10.1371/journal.pone.0165149)
Supplement: S1 Table — (DOCX) [file pone.0165149.s003.docx]

**S1 Table. Primer sets used in this study.**

| **Primer Name** | **Sequence (5’-3’)** | **Usage** |
| --- | --- | --- |
| MoAbfB_*Cla*I_F | ATCGATATGCTCTCACTCAAGGCCG | Bombardment vector construction |
| MoAbfB(w/o ss)*_Cla*I_F | ATCGATATGCAGGCGCCGGAACCCTCT |  |
| MoAbfB_*Xba*I_R | TCTAGATCCAAGTAGAATGTAGCCTCGG |  |
| MoAbfB(w/o ss)_*BamH*I_F | GGATCCCAGGCGCCGGAACCCT | Protein expression |
| MoAbfB_*Hind*III_R | AAGCTTCTCCAAGTAGAATGTAGC |  |
| MoAbfB_5UTR_*Kpn*I_F | GGTACCAATGAGACTCTGCGCTTGGATACT | Fungal mutant generation |
| MoAbfB_5UTR_*Xho*I_R | CTCGAGTTGGCATTAGCGCACGTGCTTT |  |
| MoAbfB_5L_R | CGCCCTTGCTCACCATCTTGAATTTCGTCGACGG |  |
| MoAbfB_3UTR_*Hin*dIII_F | AAGCTTCAAACCTCATGACGGGTTCC |  |
| MoAbfB_3UTR_*Spe*I_R | ACTAGTCCCATTGACCACTGCATCCTCA |  |
| MoAbfB_3L_F | GGGCAAAGGAATAGATTTTTTTTGTCTCTCTC |  |
| MoAbfB_OX_*Eco*RI_F | GAATTCTCATGCTCTCACTCAAGGCC |  |
| HYG_*Xho*I_F | AACCTCGAGCGACAGAAGATGATATTGAAGG |  |
| HYG_*Hin*dIII_Rev | CATAAGCTTCTCTAAACAAGTGTACCTGTGC |  |
| MoAbfB For | ATGCTCTCACTCAAGGCCGTGTTG |  |
| MoAbfB Rev | CTCCAAGTAGAATGTAGCCTCGGC |  |
| mCherry For | AGCAAGGGCGAGGAGGATAACATG |  |
| mCherry Rev | CTTGTACAGCTCGTCCATGCCGCCG |  |
| MoAbfB -RT-F | GCCAACTACTGCATGGGTCT | RT-PCR |
| MoAbfB -RT-R | TCTTGAACTGCGTGTTCTGG |  |
| Actin-RT-F | AGGAATGGAAGCTGCGGGTAT |  |
| Actin-RT-R | GCAGGAGGACGGCGATAACA |  |
| PR-10-RT-F | CTCGTCACGGCAATGGCATC |  |
| PR-10-RT-R | TAGGCCTCGGCGGTCTTGAA |  |
| PBZ1-RT-F | TAGCTACAGGCATCAGTGGT |  |
| PBZ1-RT-R | ACGATGTCCTTCTCCTTCTC |  |
| Cu/Zn-SOD-RT-F | ACCTGGACACCGGATCTAGC |  |
| Cu/Zn-SOD-RT-R | ATGCAACCATTGGTGGTGTC |  |
| APX1-RT-F | CTTGAGTGATCAGGACATTG |  |
| APX1-RT-R | AGCAGTAGTAGACTAGAAACCTCT |  |
| PR1-RT-F | AGTTCGTCGAGCAGGTTATCCT |  |
| PR1-RT-R | AGATTGGCCGACGAAGTTG |  |
| SLP3-RT-F | TCAACTACATCCAGGGGAAGAA |  |
| SLP3-RT-R | GTCGAAGAGCATCCATATCTCG |  |
| Duf26-RT-F | AACACCGTCTACGGCCTCCT |  |
| Duf26-RT-R | GCCTCCAGCGTGATGTCGAA |  |
| Glu2-RT-F | TCGCCTACAAAGACGACCAG |  |
| Glu2-RT-R | GATGGCGACTTATTGGGGTT |  |
